# Treatment Outcomes of Total Hip Arthroplasty Following Hip Joint Gunshot and Shell Fragment Injuries: Insights From a Single-Center Retrospective Study in Yemen

**DOI:** 10.7759/cureus.71648

**Published:** 2024-10-16

**Authors:** Abdulrakib Almirah, Anwar Mahyoub, Wael Al-Gabaly, Hatem Haidar, Wael Alhamadi, Zakarya Mothanna, Faisal Ahmed

**Affiliations:** 1 Department of Orthopedics, Faculty of Medicine, Sana'a University of Medical Sciences, Sana'a, YEM; 2 Department of Orthopedics, School of Medicine, 21 September University, Sana'a, YEM; 3 Department of Orthopedics, Faculty of Medicine, University of Science and Technology, Sana'a, YEM; 4 Department of Orthopedics, School of Medicine, Ibb University, Ibb, YEM; 5 Department of Urology, School of Medicine, Ibb University, Ibb, YEM

**Keywords:** arthroplasty., complications, gunshot wound, hip injury, operative techniques, success rate, total hip replacement

## Abstract

Background

Gunshot injuries to the hip joint are uncommon extremity injuries. The management outcomes of total hip arthroplasty for such injuries are inadequately documented and lack comprehensive treatment protocols. The purpose of this study was to evaluate the outcomes and complications associated with total hip arthroplasty following gunshot and shell fragment injury incidents in a resource-limited setting.

Materials and methods

A retrospective study of 10 patients diagnosed with hip joint gunshot and shell fragment injuries and who underwent total hip arthroplasty was conducted between November 2020 and November 2023 at the 48 Model Hospital in Sana'a, Yemen. Clinical demographic data, hip joint injury characteristics, management, complications, and outcomes were collected and analyzed. The primary end measure was the Harris Hip Score (HHS), which was calculated both before and after surgery, and the secondary outcome was the variables associated with complications.

Result

The mean age was 30.2 ± 10.7 years (range: 20-49 years). The mechanism of injury was bullets in six (60.0%) patients and shell fragments in four (40.0%). The most common previous surgery was wound debridement in eight (80.0%) patients. The mean time between fracture and total hip arthroplasty was 16.7 ± 7.5 months. The mean preoperative and postoperative HHS was 32.1 ± 8 and 69.2 ± 16.7 points, respectively. The postoperative HHS improved significantly, with a mean difference of 37.08 points (95% CI: 24.30-49.85, p = 0.002). Complications occurred in two patients (20.0%), including heterotopic ossification in one patient and surgical site infection in one. Within a mean follow-up time of 31.9 ± 11.8 months, the functional result showed poor functional outcomes in four (40.0%) patients, fair in three (30.0%), excellent in two (20.0%), and good in one (10.0%). Factors associated with postoperative complications were older age (p = 0.049), previous abdominal surgery with intestinal repair (p = 0.022), and shorter time to arthroplasty (p = 0.029).

Conclusion

In this study, total hip arthroplasty was a highly effective therapy for posttraumatic arthritis induced by bullets or shell fragments, lowering discomfort and increasing function, allowing younger patients to lead normal lives. Moreover, post-arthroplasty complications were found to be more common in patients aged older, who had previous abdominal surgery with intestinal repair, and those who underwent arthroplasty earlier.

## Introduction

Open proximal femoral fractures are a rare injury, accounting for less than 1% of femoral fractures, primarily linked to high-energy traumas like vehicular accidents, falls, or explosive incidents [1]. Nonetheless, conflict-affected areas like Yemen experience a higher prevalence of these fractures due to gunshot injuries [2]. According to the World Health Organization (WHO) and other reports, Yemen had the highest firearm-related mortality rate, at 6.1 per 100,000 people in 2016, exacerbated by civil conflict, with combat-related violence accounting for 67.2% of the excess deaths [3-5].

Shell fragments are high-energy pieces that induce severe tissue damage, whereas bullets generate just transient cavitation. As the incidence of gunshot and shell fragment wounds rises, orthopedic surgeons will face more penetrating musculoskeletal injuries [6]. Gunshot injuries to the hip joint are difficult to treat because of high fatality rates, morbidity, treatment expenses, nerve damage, and joint contamination. Furthermore, shell fragments may be associated with lead toxicity due to systemic absorption of lead from the joint milieu, lead arthropathy as a result of the interplay between irregular joint surfaces caused by fractures and the presence of intraarticular bone and lead fragments incurred during the primary traumatic event [7,8]. Bullets entering the hip joint seldom cause infection, but a transabdominal trajectory that subsequently reaches the joint raises the risk of infection following total hip arthroplasty [9]. Additionally, soft tissue injury also complicates therapy with fibrotic scar tissue formation and fibrotic toxicity hazards [10]. The pattern and severity of gunshot and shell fragments are influenced by multiple factors, including bullet trajectory, angle, velocity, and the intrinsic characteristics of the bullet, such as size and composition [11]. Other contributing factors include bullet composition, firing angle, shape, caliber, and flight characteristics, all of which affect the projectile's final velocity and the severity of the injury [12].

During the acute phase, gunshot wounds are recommended to be managed with open reduction, internal fixation, and antibiotics, along with debridement. In the chronic phase, various therapeutic strategies have been used for the management of gunshot wound-related hip injuries. However, total hip arthroplasty is an effective treatment modality in cases of resultant post-traumatic arthritis with intra-articular gunshot or shell fragments, as documented in several reports [9,13]. Heterotopic ossification, surgical site infection, and implant loosening are among the leading reasons why hip fracture patients with hip arthroplasty may require a surgical revision [14,15]. Furthermore, conversion hip arthroplasty for posttraumatic circumstances has more intraoperative and postoperative issues than total hip arthroplasty for primary osteoarthritis, especially in patients with retained implants and compromised vascularity, requiring more blood transfusions [15]. Also, these patients face higher risks of infection, transfusion need, nerve palsy, and hematoma due to greater dissection [14]. However, despite these limitations, delayed total hip arthroplasty significantly improves patients' health, highlighting the need for careful evaluation of risks and complications during patient counseling and surgical decision-making, especially for those with associated intestinal injuries.

The conflict in Yemen intensifies poverty, leading to delays in medical treatment and more fatalities. The body of research about hip arthroplasty following gunshot and shell fragment injuries is scarce, with scant documentation on its frequency, care strategies, and prognosis, especially in poor nations like Yemen [2]. This may be largely due to restricted access to healthcare facilities, a shortage of skilled surgeons, inadequate economic conditions, and no documentation, particularly in resource-constrained settings. This study aimed to assess the postoperative outcome and complications related to total hip arthroplasty after gunshot and shell fragment injuries in a resource-constrained environment.

## Materials and methods

Study design and exclusion criteria

A retrospective study was conducted at 48 Model Hospitals in Sana'a, Yemen, on 10 patients who received hip arthroplasty after gunshot and shell fragment injuries due to war-related injury to the hip joint between November 2020 and November 2023. Upon arrival at the emergency department, all patients were stabilized, antibiotics and tetanus prophylaxis were administered, and evaluated for history, physical examination, complete blood count (CBC), chemistry (blood urea nitrogen (BUN) and creatinine), and radiological assessment, including plain-radiography and computed tomography (CT) scans as well as any other assessments modalities required for additional injuries. Patients with extensive soft tissue injury around the hip, total neurologic impairment, active infection, patients with insufficient medical records or who were lost during follow-up, and those with open fractures and arterial injury that required vascular treatment (due to lack of vascular surgery in our center), and those treated at other centers were excluded.

Preoperative stage

Patients were treated using Advanced Trauma Life Support (ATLS) protocols and multidisciplinary approaches, including wound evaluation and care, until they passed the critical phase. Then, patients were scheduled as elective cases after medical and anesthesia fitness. Preoperative planning was done to manage trauma-related difficulties and degenerative changes. Blood products were prepared and transfused according to each patient's requirements.

Operative management of total hip arthroplasty and follow-up

All patients were treated with hip arthroplasty by single orthopedic senior surgeons using the posterolateral technique. Patients received preoperative prophylactic antibiotics (Cefazolin was administered for prophylaxis and maintained postoperatively until the drain was withdrawn within 48 hours) before surgery. Patients were positioned in a lateral decubitus position and under general anesthesia. The site was washed, shaved, and painted with iodine solution. Total joint arthroplasty was performed using cementless femoral and acetabular components (Zimmer Biomet, Warsaw, IN). Low molecular weight heparin was given for four weeks postoperatively. Patients were able to bear partial weight within 24 hours of surgery. After four weeks, patients were allowed full weight-bearing. Patients were released within four to six days postoperatively, provided there were no wound issues. A pelvic anteroposterior image was obtained immediately after surgery, 45 days later, at three, six, and 12 months of follow-up. Non-routine diagnostic and radiographic studies would be ordered as per the patient’s requirements. Serum lead levels would be checked in patients with retained metallic fragments, which could not be surgically removed, whether it be intra- or extra-articular. Patients were assessed using the Harris Hip Score (HHS) preoperatively and at the final follow-up examination, with a minimum postoperative follow-up time of 12 months. Furthermore, they also assessed for prosthesis infection and complications associated with an intraarticular fragment. The HHS score ranges from 0 to 100 points, with poor results defined as <70 points, fair as 70-80 points, good as 80-90 points, and excellent as 90-100 points [16].

Data gathering

The collected data include age, gender, mechanism of injury (bullet and shell fragment injury), laterality (left and right), previous surgeries (debridement and intestinal repair), preoperative and postoperative HHS, time to total hip replacement, outcome, and complications.

Main outcome

The HHS was the primary outcome measure, measured both before and after surgery at the final follow-up. The secondary outcome was to assess the variables that contributed to postoperative complications.

Statistical analysis

We employed the mean ± SD and median (IQR) for descriptive purposes for quantitative variables, while qualitative variables were characterized by their frequencies. To verify the normal distribution of the study's variables, we utilized the Shapiro-Wilk test. We used the Mann-Whitney U test for numerical data, and for categorical data, we used the chi-square test or Fisher’s exact test. Wilcoxon's test was performed to compare the difference in preoperative and postoperative HHS across all 10 patients. Differences were considered statistically significant if the p-value was less than 0.05. SPSS (IBM SPSS Statistics for Windows, IBM Corp., Version 22, Armonk, NY) was used to analyze the data.

Ethics approval

This study was approved by the 21 September University Institutional Ethics Committee (ID: S-98-H-02-F84) and followed the Helsinki Declaration. After thoroughly explaining the research purpose, all the patients consented to and signed an informed consent form for the gathering of data from their medical records, as well as the publishing of their medical information and photographs, at the time of registration.

## Results

**Figure 1:**
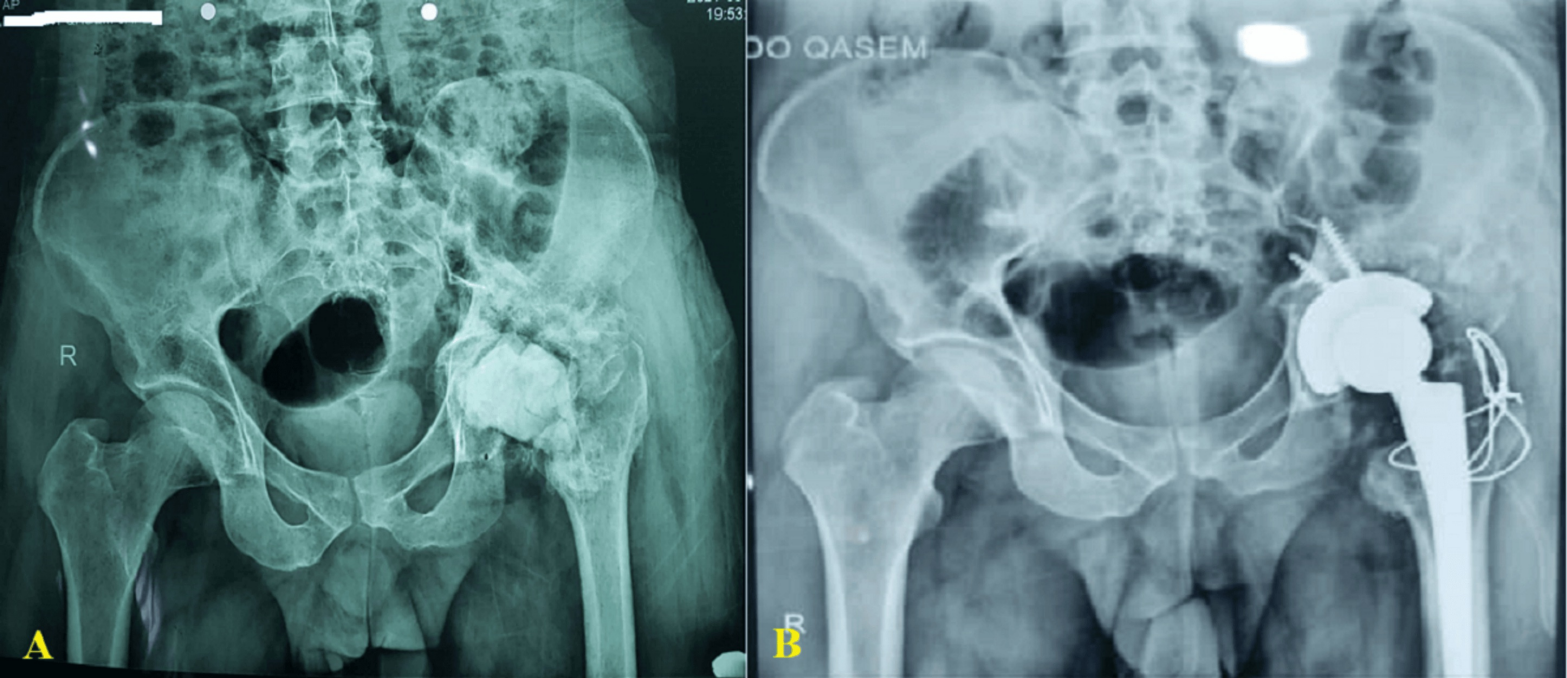
A radiologic X-ray shows (A) a 25-year-old male patient with a prior history of unsuccessful internal fixation and decreased abductor length following a gunshot wound; anteroposterior view. (B) Seven months after the accident, the patient underwent left femoral shortening osteotomy and cement-free total hip arthroplasty; anteroposterior view. Image credits: Abdulrakib Almirah, author

All patients had adequate follow-up, and none were excluded from the trial. The mean age was 30.2 ± 10.7 years, with a median of 28 years (minimum: 20 years; maximum: 49 years). The mechanism of injury was gunshot (bullet) in six (60.0%) patients and shell fragment injury in four (40.0%). Hip involvement was on the right and left side in six (60.0%) patients and four (40.0%), respectively (Figure 1).

**Figure 2:**
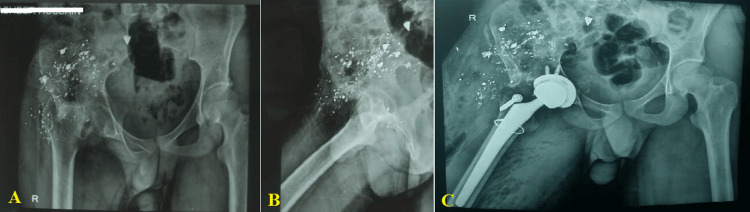
A radiologic X-ray shows (A) a 26-year-old male patient with a prior history of colostomy and contaminated hip joint after following a gunshot wound; anteroposterior view. (B) Fragment injuries within the right hip. (C) Seven months after the accident, the patient underwent right femoral shortening osteotomy and cement-free total hip arthroplasty; anteroposterior view. Image credits: Abdulrakib Almirah, author

The most common previous surgery was wound debridement with or without bone cement spacer application in eight (80.0%) cases. Other procedures were laparotomy and colostomy, with delays closer in one (10.0%) patient each (Figure2).

The mean preoperative HHS was 32.1 ± 8 points with a median of 30.1 points (minimum: 21; maximum: 43). The mean time between fracture and total hip replacement was 16.7 ± 7.5 months with a median of 21.5 months (minimum: 6 months; maximum: 23 months) (Table 1).

**Table 1:** Preoperative demographic data and fracture patterns HHS: Harris Hip Score, SD: standard deviation

Variables	Value
Age (year), mean ± SD	30.2 ± 10.7
Mechanism of injury, n (%)	
Bullet	6 (60.0%)
Shell fragment injury	4 (40.0%)
Previous surgeries, n (%)	
Debridement	8 (80.0%)
Laparotomy	1 (10.0%)
Colostomy and re-anastomosis	1 (10.0%)
Laterality, n (%)	
Right	6 (60.0%)
Left	4 (40.0%)
Preoperative HHS, mean ± SD	32.1 ± 8.0 (range: 21.0-43.0)
Time to total hip replacement (months), mean ±SD	16.7 ± 7.5 (range: 6.0-23.0)

The mean follow-up time was 31.9 ± 11.8 months with a median of 35.5 months (minimum: 9 months; maximum: 43 months). The mean postoperative HHS was 69.2 ± 16.7 points with a median of 72 points (minimum: 50, maximum: 92). Regarding the improvement of HHS score at the final follow-up, Wilcoxon’s test showed a statistically significant improvement compared to preoperative HHS (mean difference (MD): 37.08; 95% confidence interval (CI): 24.30-49.85, p = 0.002). The functional result at the last follow-up showed poor functional outcomes in four (40.0%) patients, fair in three (30.0%), excellent in two (20.0%), and good in one (10.0%) (Table 2).

**Table 2:** Postoperative data and functional outcome HHS: Harris Hip Score, SD: standard deviation

Variables	Value
Postoperative complication, n (%)	2 (20.0%)
Heterotopic ossification, n (%)	1 (10.0%)
Infection, n (%)	1 (10.0%)
Follow-up (month), mean ± SD	31.9 ± 11.8 (range: 9.0-43.0)
Postoperative HHS, mean ± SD	69.2 ± 16.7 (range: 50.0-92.0)
Functional result	
Fair, n (%)	3 (30.0%)
Excellent, n (%)	2 (20.0%)
Good, n (%)	1 (10.0%)
Poor, n (%)	4 (40.0%)

Throughout the follow-up period, no signs of lead poisoning were observed in any of the patients. The complication occurred in two (20.0%) patients, including heterotopic ossification in one patient (10.0%) who maintained normal joint mobility despite the formation of heterotopic bone and surgical site infection in one (10.0%) patient, which was managed with oral antibiotics and frequent dressings till the complete resolution of both clinical and paraclinical signs of infection.

Factors associated with postoperative complications

Factors associated with postoperative complications were older age (p = 0.049), previous abdominal surgery with intestinal repair (p = 0.022), and shorter time to arthroplasty (p = 0.029) (Table 3).

**Table 3:** Factors associated with postoperative complications in bivariate analysis IQR: interquartile range, HHS: Harris Hip Score, SD: standard deviation Statistical analysis tests used for analysis were ^1^Mann-Whitney U test and ^2^Pearson's chi-squared test. ^*^Abdominal laparotomies include previous abdominal surgery with intestinal repair in one and colostomy and re-anastomosis in one. ^†^p-values of < 0.05 were considered significant.

Variables	Subgroups	No complications (n = 8)	Complications (n = 2)	p-value^†^
Age (year)	Median (IQR)	24.0 (20.8 to 33.5)	39.0 (34.0 to 44.0)	0.049^1^
Laterality	Right	4.0 (50.0%)	2.0 (100.0%)	0.197^2^
	Left	4.0 (50.0%)	0.0 (0.0%)	
Mechanism of injury	Bullet	5.0 (62.5%)	1.0 (50.0%)	0.747^2^
	Shell fragment	3.0 (37.5%)	1.0 (50.0%)	
Previous surgery	Debridement	8 (100.0)	0 (0.0)	0.022^2^
	Laparotomy^*^	0 (0.0)	2 (100.0)	
Preoperative HHS (point)	Mean ± SD	34.8 ± 6.4 (range: 29.0-43.0)	21.5 ± 0.7 (range: 21.0-22.0)	0.895^3^
Time to arthroplasty (months)	Median (IQR)	22.0 (17.5 to 23.0)	10.0 (9.0 to 11.0)	0.029^1^
Postoperative HHS (point)	Mean ± SD	73.5 ± 15.8 (range: 50.0-92.0)	52.0 ± 2.8 (range: 50.0-54.0)	0.104^1^

## Discussion

Hip fractures resulting from gunshot and shell fragment injuries are little comprehended in the literature. These injuries are associated with the loss of bone and cartilage, which frequently hinders anatomical reduction. In addition to their destructive potential, the management of gunshot injuries presents challenges due to anecdotal experiences and a lack of evidence-based support. In this study, we analyzed the management outcome and complications of total hip arthroplasty after hip joint gunshot and shell fragment injuries in resource-limited settings. The result found that 60% of patients achieved acceptable functional outcomes, while 40% fell into poor functional outcomes, resulting in an average postoperative HHS. Furthermore, older age, previous abdominal surgery with intestinal repair, and shorter time to arthroplasty were associated with postoperative complications and subsequently worse outcomes.

High-velocity projectile injuries, resulting in open fractures, are more susceptible to infection due to extensive soft-tissue damage. These injuries require antibiotic administration, foreign body removal, irrigation, debridement, and immobilization and are treated similarly to open fractures. However, these injuries still have a higher risk of infection [6,9,17]. In a recent systematic review of total hip arthroplasty for posttraumatic osteoarthritis following acetabular fracture, Stibolt et al. found that treatment of fracture (i.e., open-reduction, internal fixation, and nonsurgical management) was reported in 44% to 100% of patients [15]. In our study, previous treatment was reported in all patients, and wound debridement was the most common procedure in 80 %. Our findings align with many previous studies assessing comparable therapies [9,10].

In this study, the mean age of victims was 29.7 ± 10.4 years, and most injuries occurred in young adults (between 27 and 49 years), with a predominance of men (100%). Previous studies, including those by Pazarci et al. and Hutaif et al., have consistently shown that most victims belong to the same age group [2,10]. A recent systematic review found that the median patient age for total hip arthroplasty was 51.5 years, with a range of 19 to 90 years [15]. The younger age in our report was predictable, as our research was conducted in a military hospital primarily serving young adult soldiers. Furthermore, we found that individuals with postoperative complications were older age than those without complications, and this difference was statistically significant. Liebergall et al. similarly discovered that patients under 40 years exhibited more favorable prognoses, likely attributable to the enhanced quality of their bone structure and the presence of fewer comorbidities [18]. Likewise, Matta et al. conducted a study of 262 acetabular fracture patients with a two-year follow-up. They found that 81% of the patients under 40 years old had good results, compared with 68% of patients aged 40 or older [19]. Furthermore, in another study, the authors noted a high rate of complications and the need for secondary arthroplasty in the first two years (up to 25%) after open reduction and internal fixation in elderly patients [20]. In contrast, other reports, such as Morison et al. and Berry et al., reported the highest revision among younger patients age [21,22]. However, in our report, this finding may be accidental, given all patients who underwent surgery were under 60 years of age. In summary, the findings suggest that patient age may have a role in the development of post-traumatic hip osteoarthritis complications.

Arthrodesis and arthroplasty should be performed as an elective procedure due to high infection risk and extensive tissue defects in open hip joint injuries during the acute phase [9]. In this study, hip arthroplasty was done in the chronic phase after gunshot and shell fragment injuries, with a mean time from injuries of 16.9 ± 7.1 months. Furthermore, our result found that individuals with postoperative complications had a shorter median time between fracture and total hip replacement than those without complications (median: 22 vs. 10 months), and this difference was statistically significant. Similarly, previous studies, including those by Pazarci et al. and Hutaif et al., have consistently shown the effectiveness of hip arthroplasty in the chronic phase [9,10,17]. A recent systematic study found that the reported duration between fracture and total hip arthroplasty ranged from 27 to 74 months, with a median length of 37 months [15]. Delayed arthroplasty provides advantages with regard to better surgical outcomes, decreased infection risk, appropriate bullet fragment extraction, and favorable bone and soft tissue healing, which results in superior patient outcomes [9,23]. Although delays in hip arthroplasty have been shown to be effective in certain settings, these studies were retrospective and lacked randomized controlled trials. A recent systematic review found no difference in functional outcomes, complications, or mortality rates between acute and delayed total hip arthroplasty groups. However, the latter was prevalent among younger ages and had a higher revision rate [24]. Future prospective research with larger sample sizes may help identify connections between time to arthroplasty and postoperative complication rates.

Due to significant bone loss from the comminution of the femoral head, reconstruction of the proximal femur is necessary. Hip arthroplasty and total hip arthroplasty are mainstays of treatment options. However, the distinction between complete hip arthroplasty and partial hip arthroplasty remains ambiguous [10]. The superiority of hip arthroplasty includes fewer dislocations and shorter surgical time. However, compared to total hip arthroplasty, it may cause more acetabular erosion [6,25]. Another hip arthroplasty surgical modality is cemented hip arthroplasty, which dramatically reduces the likelihood of intraoperative and postoperative periprostatic fractures compared to uncemented hip arthroplasty but increases surgical time and anesthetic duration. In addition, cemented fixation minimizes revisions and improves short-term results such as pain and independent walking [25,26]. The usage of newer-generation Trabecular Metal (TM) acetabular cups (Zimmer Biomet, Warsaw, IN), polyethylene liners, and interfaces may contribute to the decreased revision rate. Overall, the materials employed at the acetabular joint interface have an important role in decreasing the strain of revision [15]. Recent developments in total hip arthroplasty-bearing surface materials predict that post-traumatic hip osteoarthritis failure rates will decrease because of reduced surface wear. However, this hypothesis is constrained by short follow-up intervals, small sample sizes, and the absence of a control group [15], although cemented and uncemented hip arthroplasties vary in the method of implant fixation to the native bone. Cemented implants use bone cement, while uncemented ones use bone growth. Both systems have been proven to last over 20 years, with uncemented being preferred in younger populations under 50 and cemented in older ones over 70. In this study, we utilized uncemented total hip arthroplasty in all patients who were under 50 years, which led to excellent results and better postoperative HHS. 

In this study, the postoperative HHS was 69.2 ± 16.7 points, with poor functional outcomes in four patients (40.0%), fair in three (30.0%), excellent in two (20.0%), and good in one (10.0%). This finding was comparable to findings mentioned by Pazarci et al., which reported a mean postoperative HHS of 65.8 points, with 30% in the excellent range and 50% in the poor functional range [10]. In contrast, other reports mentioned a higher postoperative HHS score, such as Özden et al. with a postoperative HHS score of 79.92 points, Maheshwari et al. with a postoperative HHS score of 83.36 points, and Hutaif et al. with a postoperative HHS score of 78.4 points [2,9,27]. The low postoperative HHS in this study may be attributed to the nature and severity of the injuries, associated soft tissue injuries, and low preoperative HHS scores. Other reported factors for poor functional outcomes in this report may be due to high-energy trauma, infection risk, and difficult surgical procedures. Contextual issues such as inadequate resources, delayed presentations, and a lack of skilled workers may also influence the results [2]. However, instead of varied outcomes between reports, our study provides insight into the functional outcome of total hip arthroplasty after hip joint gunshot injury in resource-limited settings. Regarding the improvement of the HHS score after the operation, our result showed a statistically significant improvement. Our finding was similar to Özden et al. study [9]. In a recent systematic review containing 238 total hip arthroplasty procedures for posttraumatic osteoarthritis following acetabular fracture, the postoperative HHS increased by an average of 46.2 points (range: 41-60), resulting in a total improvement of 111% from baseline [15]. The HHS defines a severe functional limb length disparity as more than 3.2 cm, which can impact a patient's functional skills [28]. Our data indicate that no patient had a disparity surpassing this threshold.

The median incidence of complications associated with total hip arthroplasty performed in cases of posttraumatic osteoarthritis after acetabular fracture has been documented at 10.2%, with implant loosening identified as the predominant clinically significant adverse outcome, manifesting at frequencies ranging from 2% to 24%. Furthermore, the incidence rates of heterotopic ossification have also been delineated, exhibiting a spectrum from 28% to 63%, accompanied by additional stratifications according to the Brooker classification of heterotopic ossification. The documented rates of revision surgeries presented considerable variability, with such interventions occurring at rates between 2% and 32%. Iatrogenic nerve injury emerged as the least frequently reported complication, with an incidence of 3% [15]. In this study, grade one heterotopic ossification in one patient occurred, who maintained normal joint mobility despite the formation of heterotopic bone. In a recent review, Stibolt et al. mentioned that heterotopic ossification was a common complication of delayed total hip arthroplasty, with a prevalence ranging from 28% to 40% [15]. While heterotopic ossification has been documented in the literature, its occurrence and related determinants are unknown due to a lack of outcome data for such individuals. More multi-institutional research with longer follow-up periods is required to investigate heterotopic ossification incidence and determine the optimal criteria for surgical intervention and prevention.

In the first year after hip arthroplasty, revision surgery rates were 10% higher than non-traumatic total hip arthroplasty procedures, with dislocation and prosthetic joint infections being the most common reasons, and younger individuals and higher preoperative physical conditions may influence revision requirements [29]. Our study found no patient need for revision during follow-up, possibly due to the small sample size and shorter follow-up duration.

The reported infection rates following total hip replacement surgery range from 0.4% to 1.4%, with the vast majority occurring throughout the first year [27,30]. Furthermore, previous reports reveal poor functional results in cases of acetabulum fracture and pelvic contamination caused by a gunshot injury, including intestinal contents [10,30]. Özden et al. reported a higher postoperative complication rate in gunshot injuries during the Syrian civil war, with 23% of 26 patients experiencing infection postoperatively. One patient underwent resection arthroplasty, while the remaining patients underwent prosthesis removal and antibiotic-loaded cement spacer placement [9]. In another report, Maheshwari et al. reported postoperative complications following arthroplasty in three patients with gunshot injuries, including a fragmented antibiotic spacer, cement plate loosening, and wound dehiscence [27]. In this study, a case with a coinciding history of intestinal perforation that contaminated the hip joint suffered a postoperative surgical site infection following the replacement procedure, which was managed with oral antibiotics and frequent dressings till the complete resolution of both clinical and paraclinical signs of infection.

Study limitations

The main limitations of this study include a small sample size, single-center design, retrospective nature, and lack of advanced statistical analysis. Recruitment from a single urban center, primarily around the Sanaa district, may have underestimated the true incidence of hip injuries following gunshot wounds and its outcome. The methodological design, limited to retrospective chart review, is subjected to risks of selection and misclassification biases. Additionally, data accuracy may be compromised due to incomplete or inaccurate documentation. Moreover, data on important factors that significantly contribute to hip arthroplasty failures, such as smoking status, educational level, comorbidities, severity of injuries, nutritional status, or the presence of concurrent injuries, fragile health system, and medical posttreatment complications, were not recorded. Nonetheless, by giving data on the outcome of total hip arthroplasty following gunshot-related injury, our findings substantially contribute to the current approach in the literature. Furthermore, the study was done at educational hospitals in Yemen. Therefore, the results may not apply to other places. To address these limitations and give more robust results, we advocate doing a prospective comparative trial with a bigger sample size and a prolonged postoperative follow-up period.

## Conclusions

In this study, total hip arthroplasty is a highly effective therapy for posttraumatic arthritis induced by bullets or shell fragments, lowering discomfort and increasing function, allowing younger patients to lead relatively normal lives. Furthermore, patients with older age, previous abdominal surgery with intestinal repair, and shorter time to arthroplasty were associated with post-arthroplasty complications. The advantages of total hip arthroplasty for posttraumatic arthritis induced by bullets or shell fragments must be evaluated against potential hazards connected with surgery, the patient's comorbidities, the severity of injuries, and the potential risk of infection. To provide optimal contemporary care, a multidisciplinary approach should be utilized in formulating a customized treatment strategy in such complex cases, taking into account pertinent suggestions and potential risks of infections.
